# comparison between saps 3 and APACHE ii in surgical patients admitted to a brazilian ICU

**DOI:** 10.1186/2197-425X-3-S1-A836

**Published:** 2015-10-01

**Authors:** RS Nunes, EA Nicolini, MG Menegueti, MA Ferez, M Auxiliadora-Martins, A Basile-Filho

**Affiliations:** Hospital São Francisco, Ribeirão Preto, Brazil; Hospital das Clínicas da Faculdade de Medicina de Ribeirão Preto - USP, Ribeirão Preto, Brazil

## Introduction

Prognostic models are used for mortality predictions and illness severity assessment. Acute physiology and chronic health evaluation II (APACHE II) and simplified acute physiology score II (SAPS 3) are the most commonly used models worldwide. The performance of prognostic score needs to be validated before its use.

## Objectives

The aim of this study was to compare the discriminatory power of two prognostic scores, SAPS 3 and APACHE II, in surgical patients.

## Methods

Retrospectively collected data from all surgical patients admitted to a Brazilian hospital ICU between January 2011 and December 2013 were analyzed. The standardized mortality ratio (SMR) was computed for mortality prediction. The predictive ability of the APACHE II and SAPS 3 to differentiate survivors and non-survivors was determined by the ROC curve.

## Results

The data were collected of 1599 surgical patients. The mean ICU and hospital length of stay was 2.94 and 11.82 days, respectively. ICU mortality was 7.12%. Mortality predicted by customized SAPS-3 was 15.4% (SMR of 0.46) and mortality predicted by APACHE-II was 13.1% (SMR of 0.54). Discrimination was good and there was no statistically significant difference between the ROC curve of these scores: the area under the ROC curve of customized SAPS-3 was 0.716 (0.693-0.738) and the area under the ROC curve of APACHE II was 0.702 (0.679-0.725).

## Conclusions

In this group of surgical ICU patients, the performance of SAPS 3 and APACHE II were similar.

**Figure 1:**
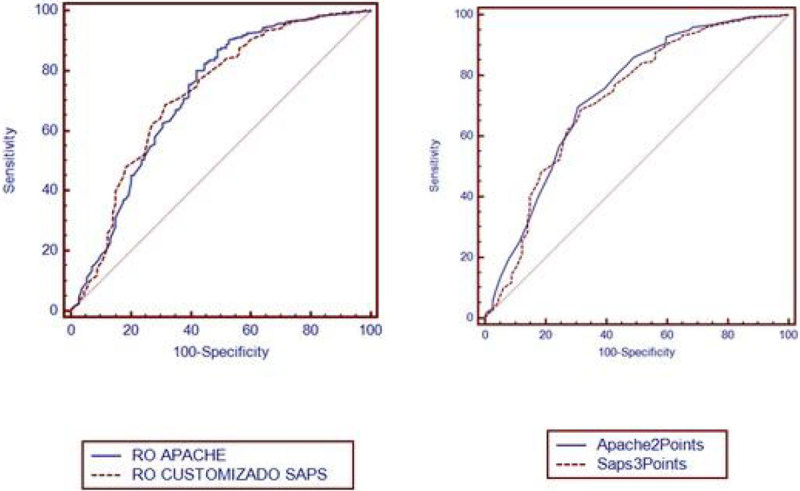
**Acute Physiology and Chronic Health Evaluation II.**

